# Autophagy deficiency exacerbates acute lung injury induced by copper oxide nanoparticles

**DOI:** 10.1186/s12951-021-00909-1

**Published:** 2021-05-31

**Authors:** Junting Xiao, Baijie Tu, Xin Zhou, Xuejun Jiang, Ge Xu, Jun Zhang, Xia Qin, Golamaully Sumayyah, Jingchuan Fan, Bin Wang, Chengzhi Chen, Zhen Zou

**Affiliations:** 1 Department of Occupational and Environmental Health, School of Public Health and Management, Chongqing Medical University, Chongqing, 400016 People’s Republic of China; 2 Key Laboratory for Biorheological Science and Technology of Ministry of Education, Chongqing University, Chongqing, 400044 People’s Republic of China; 3 Chongqing University Affiliated Cancer Hospital & Chongqing Cancer Institute & Chongqing Cancer Hospital, Chongqing, 400030 People’s Republic of China; 4 Center of Experimental Teaching for Public Health, Experimental Teaching and Management Center, Chongqing Medical University, Chongqing, 400016 People’s Republic of China; 5 Molecular Biology Laboratory of Respiratory Diseases, Institute of Life Sciences, Chongqing Medical University, Chongqing, 400016 People’s Republic of China; 6 Department of Pharmacy, The First Affiliated Hospital of Chongqing Medical University, Chongqing, 400016 People’s Republic of China; 7 Dongsheng Lung-Brain Disease Joint Lab, Chongqing Medical University, Chongqing, 400016 People’s Republic of China

**Keywords:** Copper oxide nanoparticles, Copper ions, Autophagy, *lc3b*, Acute lung injury

## Abstract

Copper oxide nanoparticles (CuONPs) are one of the widely used metal nanoparticles in the industrial and commercial fields. Autophagy is an intracellular degradation system that delivers cytoplasmic constituents to the lysosome and has been linked to nanoparticles-induced toxicity. In particular, the roles of autophagy in response to CuONPs have been explored *in vitro*, although the conclusions are controversial. To clarify the role of autophagy in CuONPs-induced acute lung injury, microtubule-associated protein 1 light chain 3 beta (*Map1lc3b* or *lc3b*) knockout mice and their corresponding wild type mice are applied. Our results showed that single-dose intratracheal instillation of CuONPs with dosages of 1.25, 2.5 or 5 mg/kg caused acute lung injury 3 days after treatment in a dose-dependent manner, as evidenced by deteriorative lung histopathology, more infiltration of macrophage cells, increased oxidative stress and copper ions. Loss of *lc3b* resulted in aggravated lung injury induced by CuONPs, which was probably due to the blockade of mitophagy and consequently the accumulation of aberrant mitochondria with overloaded copper ions. Our study provides the first *in vivo* evidence that autophagy deficiency exacerbates CuONPs-induced acute lung injury, and highlights that targeting autophagy is a meaningful strategy against CuONPs-associated respiratory toxicity.

**Supplementary Information:**

The online version contains supplementary material available at 10.1186/s12951-021-00909-1.

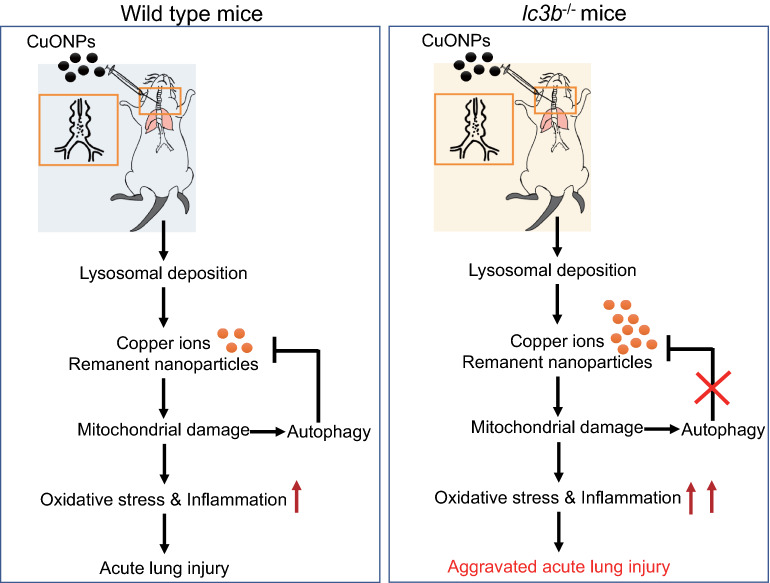

## Impact statement

Pulmonary exposure of copper oxide nanoparticles (CuONPs) are capable of inducing acute lung injury, but the underlying mechanism is obscure. The conclusions about the role of autophagy in CuONPs-induced cell death is controversial, and the *in vivo* data is lacking. This study provides the first *in vivo* evidence indicating that loss of autophagic key factor *lc3b* results in more severe acute lung injury upon CuONPs treatment, which was probably due to the blockade of mitophagy and consequently the accumulation of aberrant mitochondria with overloaded copper ions. This study may provide valuable information for the nano-bio interaction upon CuONPs exposure.

## Highlights

- Copper oxide nanoparticles (CuONPs) cause acute lung injury in a dose-dependent manner.
- CuONPs induce co-occurrence of autophagy activation and autophagic flux blockade.
- Loss of *lc3b* causes aberrant accumulation of mitochondria and excessive cooper ions.
- Loss of *lc3b* aggravates acute lung injury-induced by CuONPs treatment.

## Introduction

Owing to the excellent photovoltaic and photoconductive properties, copper oxide nanoparticles (CuONPs) have attracted great attentions and have been widely used in industrial and commercial fields such as energy storage, electrochemistry, antifouling coatings, catalysis, sensors/biosensors and biocidal agents [1]. Moreover, CuONPs have been used in biomedical devices to prevent bacterial infection due to their excellent antimicrobial characteristics [2], and have exhibited powerful potential in antitumor therapy [1, 3, 4]. The increasing production and application of CuONPs therefore have drawn attention to the potential harmful effects on human health.

Compared with SiO_2_, TiO_2_, Fe_2_O_3_ and Fe_3_O_4_ nanoparticles, CuONPs exhibit the greatest toxicity dose-dependently in many cell models [5]. The toxicity of CuONPs seems to be regulated via a Trojan horse-type mechanism, such that the nanoparticle’s form enables efficient uptake of CuONPs, and the concomitant release of excessive copper ions within the cells result in the greater toxicity [6]. The toxic effects that follow include: cytotoxic, genotoxic, and oxidative stress responses, which have been reported by our group [7–9] and other groups [3, 10]. Our previous studies also demonstrated that chelation of copper ions can significantly reverse cell death induced by CuONPs [7, 9], suggesting the crucial role of copper ions in CuONPs-induced cytotoxicity.

Mitochondrion is the key organelle involved in toxicity induced by CuONPs. Firstly, the mitochondria are considered as the most powerful intracellular source of reactive oxygen species (ROS) and are also susceptible targets for ROS assault [11]. Precise regulation of aberrant mitochondria contributes to the balance of oxidative stress. Secondly, besides lysosomes, mitochondria are considered as the major copper ions accumulation site. Once the uptake of CuONPs, the dissolution of CuONPs is prevalent in acidic milieus (for example, late endosomes and lysosomes) and consequently a profound elevation of copper ions is noted. Copper ions are transported to mitochondrial matrix for the synthesis of cupro-enzymes cytochrome c oxidase (COX) and superoxide dismutase (SOD1). Interestingly, the matrix copper pool in mitochondrion is dynamic and its size can expand, suggesting that mitochondria are feasible to sequestrate copper from the cytosol once cellular copper overload occurs [12].

Recently, autophagy dysfunction induced by nanomaterials has been recognized as a novel mechanism involved in nanomaterials-induced toxicity [13, 14]. Autophagy is an evolutionary conserved mechanism contributing to cellular homeostasis and adapts to the extracellular stress factors. Although the perturbation of autophagy process has been considered as a general phenomenon of nanoparticles, the roles of autophagy during nanoparticles exposure are controversial. The role which autophagy exerts seems to dependent on the cell types, kinds of nanoparticles and doses of exposure. In particular, CuONPs have been demonstrated to induce autophagy in endothelial cells [7–9], lung epithelial cells [10] and tumor cells [15]. Our previous results support the notion that autophagy plays a protective role during CuONPs treatment, because mitophagy (the selective autophagy for the removal of damaged mitochondria) can be activated to restore the redox status and also the copper ions balance [8].

Nevertheless, there is lack of confirmed conclusion on the role of autophagy upon CuONPs treatment. Although CuONPs exposure-induced lung injury has been reported [16, 17], the *in vivo* evidence regarding the interplay between autophagy and CuONPs-induced lung injury is absent. To this end, in the current study, microtubule-associated protein 1 light chain 3 beta (*Map1lc3b* or *lc3b*) knockout mice and their corresponding wild type mice were used to illustrate the role and possible mechanism of autophagy in CuONPs-induced lung injury. Our study will provide the first *in vivo* evidence regarding the role of autophagy in CuONPs-induced acute lung injury, therefore contributing to the in-depth understanding of CuONPs-associated pulmonary toxicity.

## Results

### Characteristic of copper oxide nanoparticles (CuONPs)

**Fig. 1:**
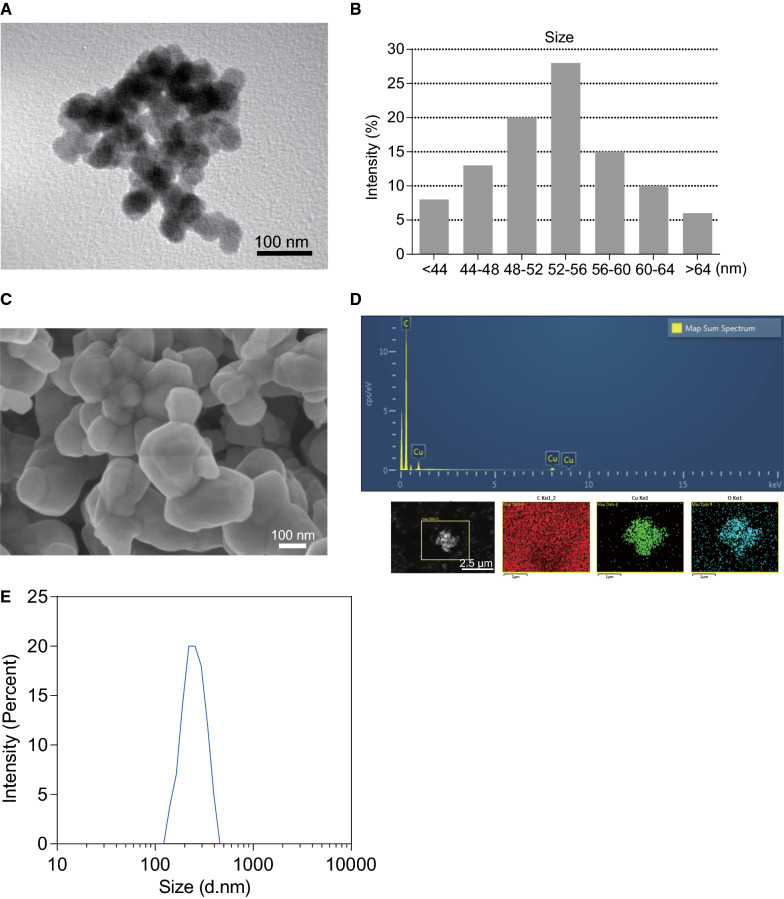
Physical-chemical characteristics of CuONPs used in this study. **A** The TEM image of CuONPs. Note that the CuONPs were spheroid or cubic. Aggregation of CuONPs was observed. Scale bar, 100 nm. **B** The diameter of CuONPs was calculated according to the TEM results. Note that the size of CuONPs was about 50 nm. **C** The SEM image of CuONPs. Scale bar, 100 nm. **D** FE/SEM-EDS was applied to determine the composition of CuONPs. **E** The hydrodynamic diameters of CuONPs were determined by DLS analysis

The size and morphology of the CuONPs were characterized by transmission electron microscopy (TEM). As shown in the TEM image, CuONPs used in this study was spherical or cubical (Fig. 1A), and the average diameter of the CuONPs was about 50 nm (Fig. 1B). Scanning electron microscope (SEM) further showed that the spherical or cubical shape of CuONPs and indicated the aggregation of CuONPs (Fig. 1C). To further determine the characteristics of CuONPs, FE/SEM-EDS was employed for elemental analysis. As depicted in Fig. 1D, there were specific peaks that correspond to Cu and O elements in the image, respectively. Moreover, according to the results of dynamic light scattering (DLS), the average hydrodynamic diameter of CuONPs in suspension solution was 252.8 nm (Fig 1E) and the zeta-potential was − 16.3 mV. The hydrodynamic diameter of CuONPs was much larger than the average size (about 50 nm) obtained from TEM observation. This discrepancy might be due to the formation of the hydration layer of the nanoparticles or aggregation in suspension solution.

### Intratracheal instillation of CuONPs induced acute lung injury in mice

**Fig. 2:**
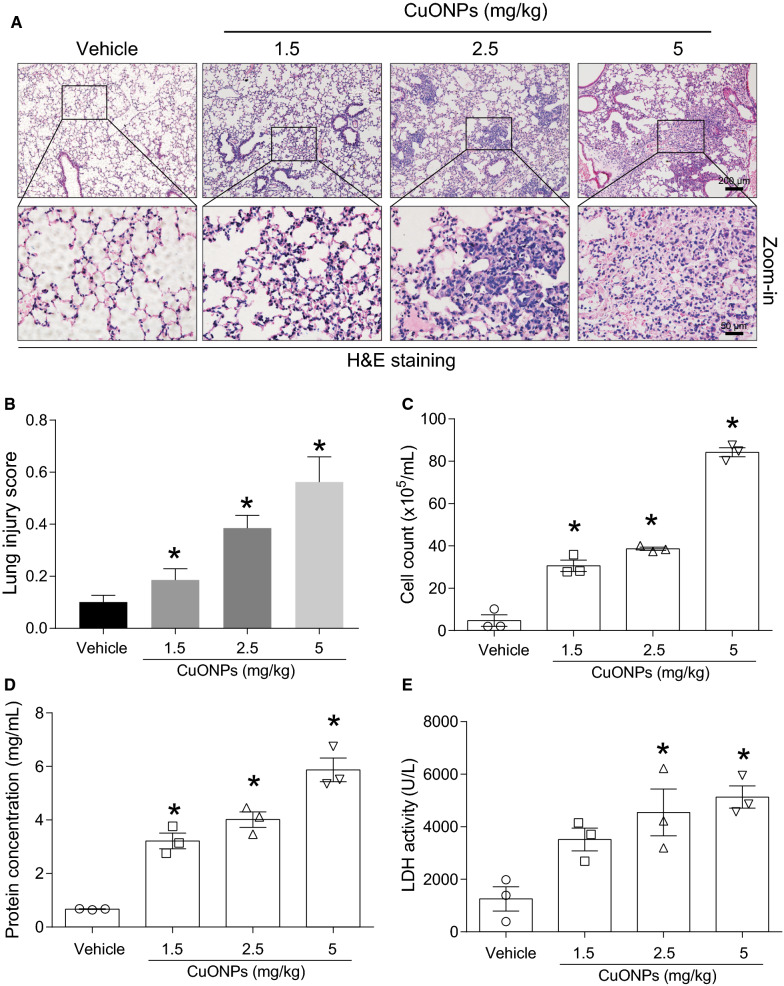
CuONPs instillation caused acute lung injury dose-dependently in mice. **A** Representative H&E staining images of lung tissues of mice 3 day after intratracheally administration of vehicle, 30, 50 or 100 μg CuONPs, respectively. Scale bar, 200 μm in the upper panels and 50 μm in the bottom panels. **B** The lung injury scores of H&E staining were calculated. **C** The cell counts in BALFs collected from indicated groups were determined by automated cell counter. **D** The protein concentrations of BALF collected from indicated groups were determined by BCA analysis. **E** The LDH activities of BALFs collected from indicated groups were determined. *indicates *p *< 0.05 compared with vehicle group

Our previous studies [7, 8] and other study [10] have reported that CuONPs cause cell death in endothelial cells and lung alveolar cells. Herein, we established an animal model showing that CuONPs cause acute lung injury in mice by a single dose intratracheal instillation at dosages of 1.5, 2.5 and 5 mg/kg, respectively. 3 days after instillation, mice lung tissues and bronchoalveolar lavage fluid (BALF) in the indicated groups were obtained for lung injury assessment. Histopathological results from hematoxylin and eosin (H&E) staining showed that CuONPs instillation induced dose-dependent lung tissue injuries, including infiltration of inflammatory cells, increased thickness of the alveolar wall and disruption of alveolar structure (Fig. 2A). The lung injury scores calculated from the H&E staining also exhibited an elevated tendency in response to CuONPs treatment (Fig. 2B). The cell counts and protein concentration in BALF are typically recognized as the markers for increased alveolocapillary membrane permeability and inflammation. CuONPs instillation significantly increased cell counts (Fig. 2C) and protein concentration (Fig. 2D) in BALF. Lactate dehydrogenase (LDH), a soluble cytosolic enzyme is released into the extracellular milieu when disruption of plasma membrane or cell death occurs [18]. We found that CuONPs instillation significantly upregulated LDH in the BALF (Fig. 2E), suggesting the possible damage of pulmonary epithelial cells upon CuONPs treatment.

**Fig. 3:**
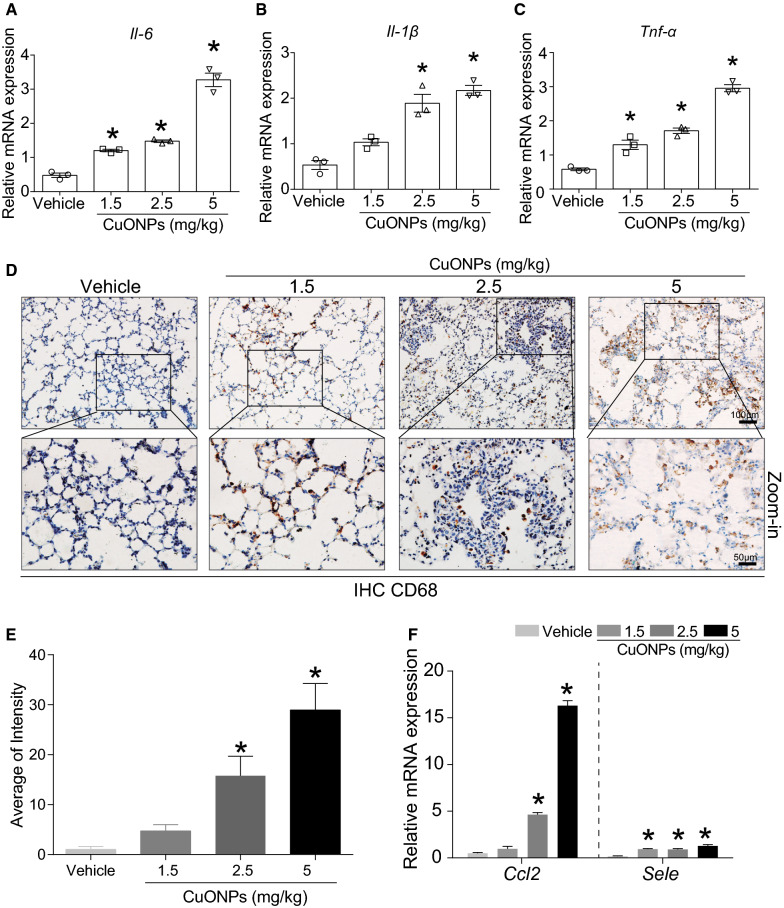
CuONPs instillation caused inflammation in lung tissues of mice. Mice were intratracheally instilled with vehicle, 1.5, 2.5 or 5 mg/kg CuONPs, respectively for 3 days. The mRNA expression level of **A** *Il-6*, **B** *Il-1β* and **C** *Tnf-α* in mice lung tissues in the indicated groups were determined by QPCR analysis. **D** The expression levels of CD68 in lung tissues in the indicated groups were determined by IHC analysis. Scale bar, 100 μm in the upper panels and 50 μm in the bottom panels. **E** The average of CD68 intensity shown in **D** were quantified. **F** The mRNA expression levels of *Ccl2* and *Sele* in lung tissues in the indicated groups were determined by QPCR assay. *indicates *p *< 0.05 compared with vehicle group

Moreover, CuONPs instillation also increased the mRNA expression levels of proinflammatory factors, such as interleukin 6 (*Il-6)* (Fig. 3A), interleukin-1β (*Il-1β*) (Fig. 3B) and tumor necrosis factor-α (*Tnf-α)* (Fig. 3C). Proinflammatory cytokines are a general term for those immunoregulatory cytokines that facilitate inflammation. Proinflammatory cytokines are produced predominantly by activated macrophages and are involved in the up-regulation of inflammatory reactions. In particular, IL-1β, TNF-α, and IL-6 are recognized as the major proinflammatory cytokines [19]. TNF-α binds to two specific receptors on the cell membrane, TNF receptor I and TNF receptor II, and exhibits both the proinflammatory and immunoregulatory properties of cytokines. IL-1β is a pleiotropic and immunoregulatory cytokine. IL-1 family consists of two major agonistic proteins called IL-1α and IL-1β and one IL-1 receptor antagonist. IL-6 is a pleiotropic cytokine produced in response to inflammatory stimuli [20]. The elevation of IL-6, TNF-α, and IL-1β in the lung tissues of mice-treated with CuONPs resulted in augmentation of inflammation and contribution of lung injury caused by CuONPs treatment. CD68 is a pan-macrophage/dendritic cell marker. It recognizes alveolar macrophages in naïve status and also stains immigrant monocytes in infected status [21]. Immunohistochemistry (IHC) staining results showed that infiltrations of CD68-positive cells were prevalent (Fig. 3D), and the intensity of CD68-positive cells was profoundly elevated in the lung tissues of CuONPs-treated mice (Fig. 3E). *Ccl2* (C-C motif chemokine ligand 2) encodes CCL2 (or MCP-1), which can recruit monocytes, and dendritic cells to inflammatory sites in the status of either tissue injury or infection [22]. *Sele* gene encodes E-selectin (or CD62E) protein, mainly contributes in the regulation of the initiation of adhesion process and facilitates leukocyte rolling and adhesion [23]. We found that the mRNA expression levels of *Ccl2* and *Sele* in mice lung tissues were elevated upon CuONPs treatment (Fig. 3F), suggesting recruitment of circulating monocytes to the inflammatory lung tissues.

**Fig. 4:**
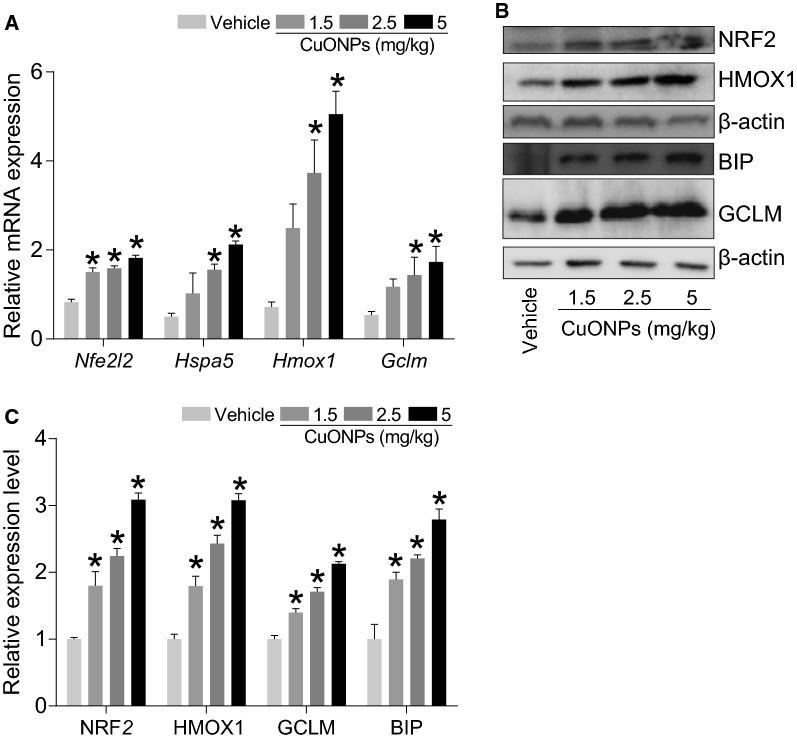
CuONPs instillation caused oxidative stress in lung tissue of mice. Mice were intratracheally instilled with vehicle, 1.5, 2.5 or 5 mg/kg CuONPs, respectively for 3 days. **A** The mRNA expression levels of *Nfe2l2*, *Hspa5*, *Hmox1* and *Gclm* in mice lung tissues in the indicated groups were determined by QPCR analysis. **B** The protein expression levels of NRF2, HMOX1, GCLM and BIP in mice lung tissues in the indicated groups were determined by western blot analysis. β-actin was served as internal control. **C** Quantification of the relative protein expression levels shown in **B** *indicates *p *< 0.05 compared with vehicle group

In addition, we found that CuONPs instillation can remarkably increase the mRNA expression level of anti-oxidative genes *Nfe2l2*, *Hspa5*, *Hmox1* and *Gclm* (Fig. 4A) and their corresponding protein NRF2, BIP, HMOX1 and GCLM expression levels (Fig. 4B, C), indicating that oxidative stress was induced by CuONPs instillation.

Overall, the above results indicated that CuONPs instillation is capable of triggering acute lung injury, which is mainly manifested by lung histopathological deterioration, inflammation and oxidative stress.

### CuONPs instillation led to elevation of copper ions in lung tissues of mice

**Fig. 5:**
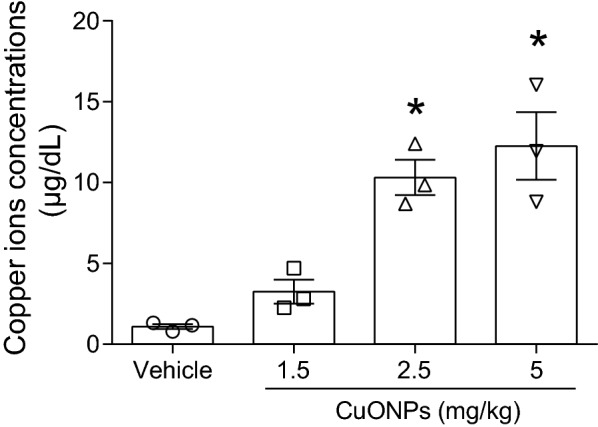
CuONPs instillation caused elevation of copper ions in lung tissue of mice. Mice were intratracheally instilled with vehicle, 1.5, 2.5 or 5 mg/kg CuONPs, respectively for 3 days. The concentrations of copper ions in the mice lung tissues were determined. *indicates *p *< 0.05 compared with vehicle group

In previous studies conducted by our group, we illustrated that lysosomal deposition of CuONPs can result in the dissolution of CuONPs and the release of copper ions. Chelation of copper ions significantly alleviated cell death induced by CuONPs [7]. Our results highlight the importance of copper ions in the toxicity induced by CuONPs *in vitro*, which agree with other studies. Herein, the copper ions in the lung tissues after CuONPs instillation were determined. The results indicated that CuONPs instillation increased the copper ions concentration in the lung tissue of mice (Fig. 5), hinting that CuONPs-induced acute lung injury is associated with copper ions released from CuONPs.

### Autophagy process was influenced by CuONPs instillation in the lung tissues of mice

**Fig. 6:**
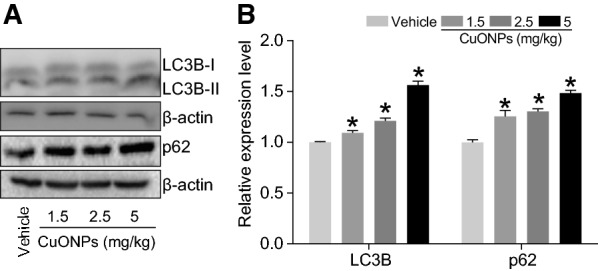
CuONPs instillation caused autophagy dysfunction in lung tissue of mice. **A** Mice were intratracheally instilled with vehicle, 1.5, 2.5 or 5 mg/kg CuONPs, respectively. After 3 days, the protein expression levels of LC3B-I/II and p62 in the mice lung tissues were determined by western blot analysis. β-actin was served as internal control. **B** Quantification of the relative protein expression levels shown in (**A**). *indicates *p *< 0.05 compared with vehicle group

**Fig. 7:**
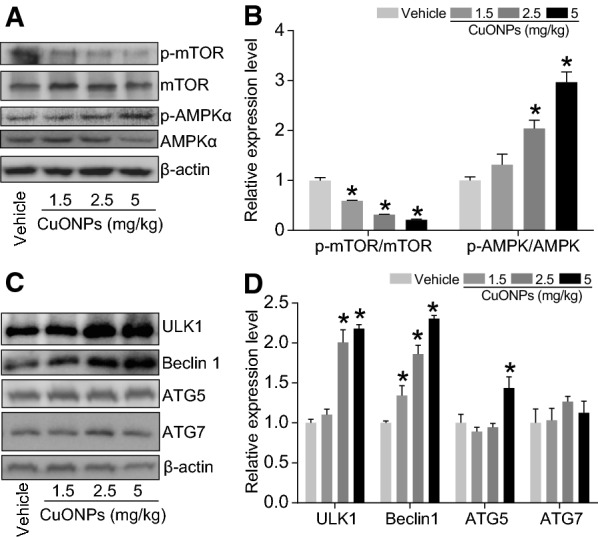
Autophagy-related protein changed upon CuONPs instillation in lung tissue of mice. **A**, **B** Mice were intratracheally instillation with vehicle, 1.5, 2.5 or 5 mg/kg CuONPs, respectively. After 3 days, the protein expression levels of p-mTOR, mTOR, p-AMPKα and AMPKα in the lung tissues were determined by western blot analysis. β-actin was served as internal control and the indicated protein expression levels were quantified. **C**, **D** The protein expression levels of p-mTOR, mTOR, p-AMPKα and AMPKα in the lung tissues were determined by western blot analysis. β-actin was served as internal control and the indicated protein expression levels were quantified. *indicates *p *< 0.05 compared with vehicle group

In the previous studies, we revealed that lysosomal deposition of CuONPs resulted in the damage of lysosome, and consequently the dysfunction of autophagy, suggesting that autophagy might play a protective role against cytotoxic effects induced by CuONPs [7, 8]. In the current study, the results demonstrated that the elevation of LC3B-II/I ratio (a classic autophagy maker) and the increase of p62/SQSTM1 (the substrate of autophagy) in lung tissues of mice after CuONPs treatment (Fig. 6A, B). Key factors involved in autophagy machinery were further determined. We found that CuONPs treatment caused a decrease in p-mTOR expression level and an increase in p-AMPKαexpression level (Fig. 7A, B), which might contribute to the upregulation of LC3B/II-I. Consequently, the downstream factors of mTOR/AMPK signaling pathway, such as ULK1, Beclin1 and ATG5, were all upregulated significantly by CuONPs treatment in the lung tissues of mice. Intriguingly, the expression level of ATG7 was not profoundly changed upon CuONPs treatment (Fig. 7C, D). The scenario of autophagy machinery in *in vivo* model is consistent with that *in vitro* cell model after CuONPs treatment.

### Loss of lc3b aggravated acute lung injury induced by CuONPs instillation

**Fig. 8:**
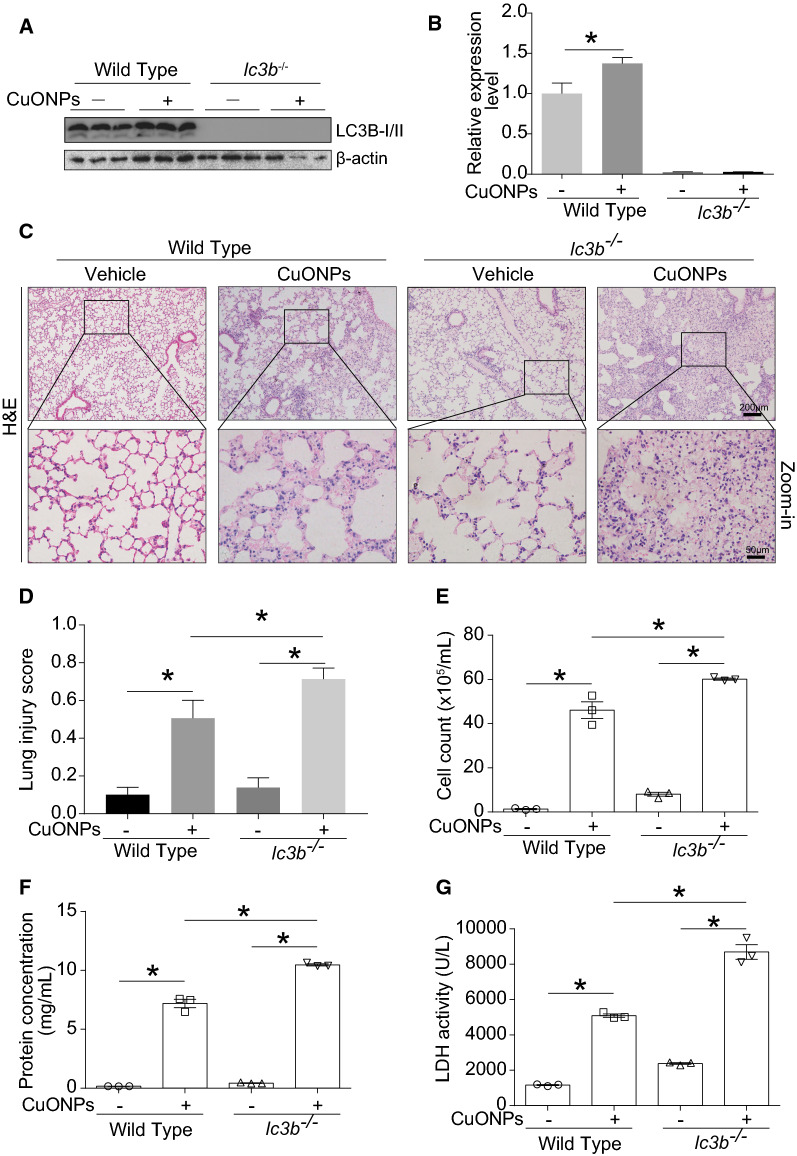
Loss of *lc3b* aggravated lung injury induced by CuONPs. Wild type and *lc3b*^-/-^ mice were intratracheally instilled of vehicle or 5 mg/kg CuONPs, respectively, then the lung tissues and BALFs of indicated groups were collected 3 days after treatment. **A**, **B** The protein expression levels of LC3B-I/II in the mice lung tissues were determined by western blot analysis. β-actin was served as internal control and the indicated protein expression levels were quantified. **C** Representative H&E staining images of lung tissues. Scale bar, 200 μm in the upper panels and 50 μm in the bottom panels. **D** The lung injury scores of H&E staining were calculated. **E** The cell counts in BALFs collected from indicated groups were determined by automated cell counter. **F** The protein concentrations of BALF collected from the indicated groups were determined by BCA analysis. **G** The LDH activities of BALFs collected from indicated groups were determined. *indicates *p *< 0.05 compared with indicated group

*lc3b* is the key gene for autophagy/mitophagy, and the loss of *lc3b* would result in autophagic deficiency [24]. Results of western blot analysis showed that CuONPs instillation led to the increase of LC3B-II/I in lung tissue of wild type mouse, while LC3B protein disappeared in the lung tissue of *lc3b*^*-/*-^ mice with or without CuONPs instillation (Fig. 8A, B). Histopathology results exhibited more severe lung injury in *lc3b*^*-/*-^ mice in comparison with wild type mice after CuONPs treatment (Fig. 8C, D). The cell counts (Fig. 8E) and protein concentrations (Fig. 8F) in BALF of *lc3b*^*-/*-^ mice were significantly elevated in comparison with wild type mice when expose to CuONPs, whereas there was unapparent change between *lc3b*^*-/*-^ mice and wild type mice without CuONPs treatment. The similar tendency was also observed in the LDH activity (Fig. 8G), suggesting the possibility that CuONPs instillation caused more severe lung epithelial cell damage in *lc3b*^*-/*-^ mice in comparison with wild type mice.

**Fig. 9:**
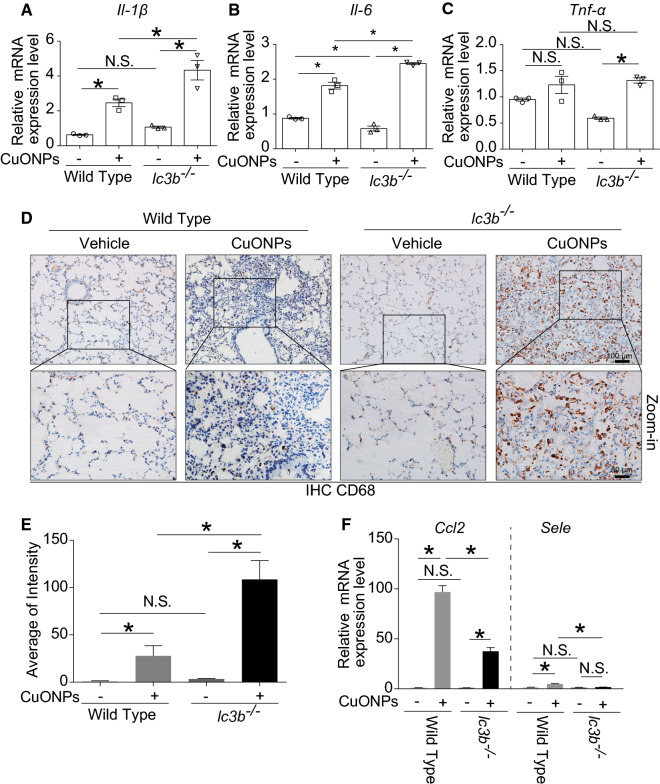
Loss of *lc3b* aggravated pulmonary inflammation induced by CuONPs. Wild type and *lc3b*^-/-^ mice were intratracheally instilled of vehicle or 5 mg/kg CuONPs, respectively, then the lung tissues of indicated groups were collected 3 days after treatment. The mRNA expression levels of **A** *Il-1β*, **B** *Il-6* and **C** *Tnf-α* in mice lung tissues in the indicated groups were determined by QPCR analysis. **D** The expression levels of CD68 in mice lung tissues in the indicated groups were determined by IHC analysis. Scale bar, 100 μm in the upper panels and 50 μm in the bottom panels. **E** The average of CD68 intensity shown in (**D**) were quantified. **F** The mRNA expression levels of *Ccl2* and *Sele* in mice lung tissues in the indicated groups were determined by QPCR analysis. *indicates *p *< 0.05 compared with indicated group

Regarding to the inflammatory response, loss of *lc3b* gene resulted in higher elevation of mRNA expressions of *Il-1β* (Fig. 9A) and *Il-6* (Fig. 9B), while not *Tnf-α* (Fig. 9C) in lung tissues of mice exposed to CuONPs treatment. Consistently, loss of *lc3b* gene also caused more infiltration of CD68-positive macrophage cells in lung tissues (Fig. 9D, E). Interestingly, the mRNA expressions of *Ccl2* and *Sele* were significantly decreased in *lc3b*^*-/*-^ mice in comparison with wild type mice treated with CuONPs (Fig. 9F), signifying that the chemotaxis was downregulated when *lc3b* was lost.

**Fig. 10:**
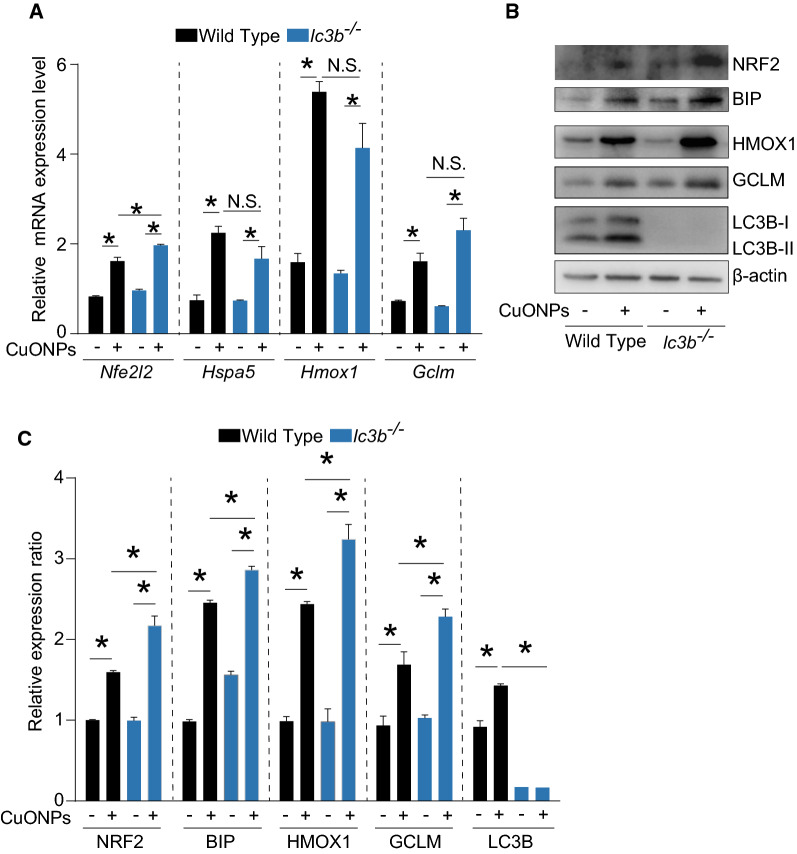
Loss of *lc3b* aggravated pulmonary oxidative stress induced by CuONPs. Wild type and *lc3b*^-/-^ mice were intratracheally instilled of vehicle or 5 mg/kg CuONPs, respectively, then the lung tissues of indicated groups were collected 3 days after treatment. **A** The mRNA expression levels of *Nfe2l2*, *Hspa5*, *Hmox1* and *Gclm* in mice lung tissues in the indicated groups were determined by QPCR analysis. **B** The protein expression levels of NRF2, HMOX1, BIP and GCLM in mice lung tissues in the indicated groups were determined by western blot analysis. β-actin was served as internal control. **C** Quantification of the relative protein expression levels shown in (**B**) *indicates *p *< 0.05 compared with indicated group

The key factors involved in oxidative stress were also determined. Our data showed that only the mRNA expression level of *Nfe2l2* was slightly upregulated in *lc3b*^*-/*-^ mice in comparison with wild type mice in response to CuONPs instillation, however the mRNA expression levels of *Hspa5*, *Hmox1* or *Gclm* were not changed significantly (Fig. 10A). Strikingly, we found that CuONPs instillation profoundly increased the protein expression levels of NRF2, GCLM, BIP and HMOX1 (Fig. 10B, C) in *lc3b*^*-/*-^ mice in comparison with wild type mice. These data together suggest that loss of *lc3b* has significant influence on the key factors involved in oxidative stress-caused by CuONPs.

**Fig. 11:**
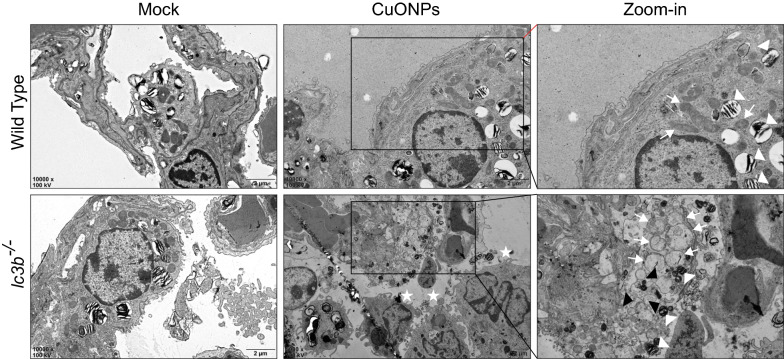
Loss of *lc3b* led to more severe deteriorative pulmonary ultrapathological changes induced by CuONPs. Wild type and *lc3b*^-/-^ mice were intratracheally instilled of vehicle or 5 mg/kg CuONPs, respectively, then the lung tissues of indicated groups were collected 3 days after treatment. The ultrastructural pathology of lung tissues were determined by TEM. Scale bar, 2 μm. Note that the white stars indicate macrophage cells; the white arrows indicate mitochondria; the white triangles indicate lamellar corpuscle, which were the marker of type II alveolar epithelial cells; the black triangles indicate the dilatation and vesiculation of endoplasmic reticulum

**Fig. 12:**
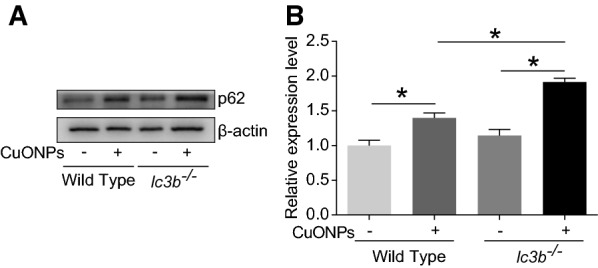
Loss of *lc3b* led to profound elevation of p62 in CuONPs-treated mice lung tissues. Wild type and *lc3b*^-/-^ mice were intratracheally instilled of vehicle or 5 mg/kg CuONPs, respectively, then the lung tissues of indicated groups were collected 3 days after treatment. The protein expression levels of p62 in mice lung tissues in the indicated groups were determined by western blot analysis. β-actin was served as internal control. **B** Quantification of the relative protein expression levels shown in (**A**) *indicates *p *< 0.05 compared with indicated group

Ultrahistopathological results from images of TEM (Fig. 11) showed that loss of *lc3b* caused more infiltration of macrophage cells (the white stars) in the alveolus. In the zoom-in picture of TEM image of type II alveolar epithelial cells (the white triangles, indicated lamellar corpuscle, which are the marker of type II alveolar epithelial cells), aberrant mitochondria (the white arrows) and endoplasmic reticulum with dilatation and vesiculation (the black triangles) were prevalent in *lc3b*^*-/*-^ mice, indicating that loss of *lc3b* was associated infiltration of inflammatory cells and accumulation of aberrant mitochondria and endoplasmic reticulum. The plausible reason was likely that loss of *lc3b* dampened the autophagy process, as evidenced by the more upregulation of p62 expression level in the lung tissues of *lc3b*^*-/*-^ mice as comprised with wild type mice after CuONPs treatment (Fig. 12A, B).

### Loss of lc3b led to higher copper ions accumulation in the lung tissue upon CuONPs treatment

**Fig. 13:**
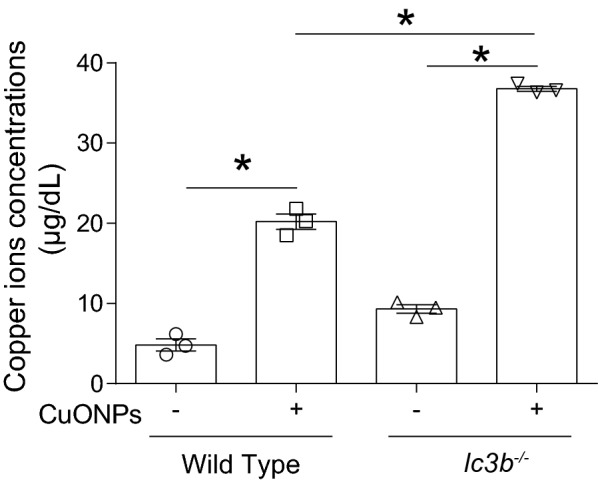
Loss of *lc3b* caused higher release of copper ions in lung tissue of mice upon CuONPs treatment. Wild type and *lc3b*^-/-^ mice were intratracheally instilled of vehicle or 5 mg/kg CuONPs, respectively, then the lung tissues of indicated groups were collected 3 days after treatment. The concentrations of copper ions in the lung tissues were determined. *indicates *p *< 0.05 compared with indicated group

As mentioned above, the release of copper ions is critical for the toxicity induced by CuONPs. Therefore, the copper ions in the lung tissue were assessed. The results showed that loss of *lc3b* can lead to more profound elevation of copper ions in the lung tissues in comparison with wild type mice after CuONPs treatment (Fig. 13), suggesting that loss of *lc3b* was closely linked to the increase of copper ions in the lung tissue of mice.

## Discussion

Accumulating evidence indicates that autophagy perturbation is well correlated with toxicity induced by metal nanoparticles [13, 14, 25, 26]. Our previously published data have illustrated the mechanism regarding the correlation between autophagy process and CuONPs-induced toxicity in cell model. We found that CuONPs can enter into cell cytoplasm via endocytosis, and were subsequently delivered to lysosomes, which are the central component for intact autophagic flux [7]. In the acidic environment of lysosomes (pH ranging from ~ 4.5–5.0) [27], the CuONPs with particle forms are possibly dissolved into copper ions form, although possible remnant of particle form of CuONPs may exist. The combination effect of copper ions and remnant of CuONPs together caused lysosomal and mitochondrial damage [8]. Consequently, lysosomal damage further induced deficiency of autophagic flux, in particular the mitophagy, which is the major approach for the removal of damaged mitochondria within the cells. Incomplete mitophagy also contributes to excess oxidative stress and ultimately the cell death. Despite knockout of ATG5 is able to exacerbate cell death induced by CuONPs *in vitro*, the application of chelator for copper ions displays the opposite effect [8]. Our results suggest that exposure of CuONPs can perturb the autophagy process and cause copper ions-dependent toxicity *in vitro* cell model, and highlight that autophagy plays a protective role against CuONPs toxicity in endothelial cell. It is noteworthy that there are controversial opinions on the role of autophagy upon CuONPs treatment in different cell models. For instance, in human breast cancer cells MCF7, CuONPs exposure can induce autophagy, which serves as a protection mechanism against apoptosis [28]. In contrast, another study declared that CuONPs induced excess autophagy in MCF7 cells, and the role of autophagy was to cause a form of cell death named as autosis [15]. In human type II alveolar epithelial cell line (A549), it was reported that CuONPs triggered autophagic cell death, and pharmacological inhibition of autophagy with 3-MA and wortmannin (both are classic autophagy inhibitors) effectively attenuated cell death induced by CuONPs. The role of autophagy in CuONPs-induced A549 cytotoxicity was likely to promote the cell death [10]. Up to now, the data from *in vivo* model regarding the role of autophagy in CuONPs-induced acute lung injury is still lacking.

The current study was aimed to illustrate the possible role of autophagy in CuONPs-induced acute lung injury. We used a single-dose intratracheal instillation of CuONPs at dosages of 1.5, 2.5 and 5 mg/kg, respectively. The single-dose intratracheal instillation has several advantages. Firstly, intratracheal instillation can ensure accurate exposure dosage at the greatest degree in comparison with nasal instillation or systemic exposure, since intratracheal instillation can directly deliver CuONPs into lower respiratory tract or pulmonary alveoli. Secondly, intratracheal instillation is easy to perform and stable. Additionally, the single-dose instillation can only induce the minimal damage. Therefore, intratracheal instillation is broadly used for pulmonary toxicity test in experimental animals. Since the background of *lc3b* KO mice is C57BL/6J, therefore we chose C57BL/6J mice to perform this study. Traditionally, male mice are usually used for toxicity studies unless specific demands. It is also interesting to further investigate whether female mice have the same characteristics as male mice when exposed to CuONPs in our future study. Regarding to the choice of exposure time, we referred to our preliminary study, which showed that CuONPs instillation caused lung injury 1 day after exposure, and reached to the peak 3 days after exposure.

Herein, we firstly demonstrated that CuONPs instillation can induce acute lung injury in rodent’s animal in a dose-dependent manner, as evidenced by the deteriorated pathology, inflammation and oxidative stress in lung tissues of mice 3 days after exposure (Figs. 2, 3, 4). Our results are well consistent with previous studies [16, 17]. Beyond the acute inflammatory response, CuONPs exposure was also able to induce accumulation of collagen and α-SMA in the lung tissues [17], which subsequently led to fibrosis [29]. However another study reported that no fibrotic changes were detected 4 weeks after CuONPs instillation [16]. This discrepancy may be due to the differences in the type of CuONPs, animal model, exposure means, or dosage used. Nevertheless, these chronic phenomena, to a large extent, are correlated with inflammation and oxidative stress induced by CuONPs in the acute phase [30].

To our knowledge, although autophagy is reported to be involved in CuONPs-induced cytotoxicity in multiple cell lines, scarce studies focused on the relationship between autophagy and acute lung injury in the context of CuONPs treatment. Based on the above established CuONPs-induced acute lung injury mice model, the alteration of autophagy process was determined upon CuONPs instillation. Consistent with our *in vitro* results, we found that CuONPs treatment activated autophagy machinery, as evidenced by elevation of LC3B-II/I ratio, ULK1, Beclin 1 and ATG5. Interestingly, we failed to observe the elevation of ATG7 after CuONPs treatment, suggesting the selectivity of activation of autophagy branch (Figs. 6, 7). The activation of autophagy machinery are probably due to the decrease of p-mTOR and increase of p-AMPKα (Figs. 6, 7), which are the crucial upstream factors for autophagy regulation [31]. In addition, the autophagy substrate p62 was upregulated upon CuONPs treatment, implying that autophagic flux was dampening. All these data from *in vivo* evidence presented in this study and *in vitro* models [7] reported previously together depict the scenario that the co-occurrence of the activation of autophagy machinery and blockade of autophagic flux after CuONPs treatment.

The role of fluctuation of autophagy machinery was further investigated in *lc3b* gene knockout mice (*lc3b*^-/-^) model and their corresponding wild type mice. There is almost no LC3B protein detected with or without CuONPs treatment, and the absence of *lc3b* gene profoundly exacerbated lung injury, proinflammatory cytokines expression and infiltration of macrophage (Figs. 8, 9). We inferred that loss of *lc3b* induced deteriorative lung injury in mice might due to the retardation of autophagic degradation of aberrant mitochondria, as evidenced by TEM and western blot results (Figs. 10, 11). As mentioned above, mitochondria contain two enzymes, COX and SOD1, which need copper as a cofactor to sustain their biological activity. The copper is likely originating from a conserved, bioactive mitochondrial matrix copper pool. Once the cellular copper overload occurs, the size of mitochondrial matrix copper pool can expand to adapt the change of copper ions, thus the mitochondria will serve as container for excessive copper [12, 32–34]. Actually, abundant copper ions were found in mitochondria, besides lysosomes. It is possible that accumulation of aberrant mitochondria would result in elevated copper ions, which are the crucial factor for determining toxicity of CuONPs. To verify this hypothesis, the copper ions level in lung tissues of mice were assessed. As expected, CuONPs instillation significantly elevated the copper ions dose-dependently (Fig. 5). Ilse Gosens et al. reported that around 2% of CuO was present as copper ions in Gamble’s solution (pH 7.4), but in artificial lysosomal fluid (pH 4.5), 60% of CuONPs rapidly dissolved within 1 h [16]. These data together indicate that CuONPs dissolve into copper ions may be the basis of CuONPs-associated toxicity. More importantly, the loss of *lc3b* resulted in higher elevation of copper ions in lung tissues of mice (Fig. 13), implying that *lc3b* is essential for homeostasis of copper ions upon CuONPs treatment. These above results together suggest that the loss of *lc3b* will result in the blockade of mitophagy, and consequently, leading to the accumulation of aberrant mitochondria and excessive copper ions, all of which further aggravate the lung injury induced by CuONPs.

In the previous study, we reported that the blockade of mitophagy during CuONPs exposure can obviously enhance cytotoxicity through production of mitochondria-originated excessive oxidative stress [8]. Although the mRNA expression levels of the key factors involved in regulation of oxidative stress were equal in *lc3b*^*-/-*^ mice and wild type mice treated of CuONPs, the protein expression level of NRF2, BIP, HMOX1 and GCLM was further increased in *lc3b*^*-/-*^ mice exposed to CuONPs (Fig. 10), suggesting that the enhanced oxidative stress caused by autophagy deficiency is not due to the elevation of transcriptional activity of key factors involved in antioxidative mechanisms. To our surprise, the mRNA expression levels of *Ccl2* and *Sele* were profoundly reduced in *lc3b*^*-/-*^ mice (Fig. 9F), suggesting that the decreased chemotactic activity when *lc3b* was lost. We speculated these phenomena were probably due to the complexity of lung tissue as compared with *in vitro* cell models. Upon excessive lung tissue inflammation and injury, other protective mechanisms, besides autophagy, might also be activated to restore the balance in lung tissues. There is still a certain possibility that the lung injury caused by CuONPs instillation will recover with time. For example, it was reported that exposure equivalent to an aerosol of agglomerated CuONPs caused toxicity in rats dose-dependently, however the toxic effects almost completely resolved 21 days after exposure [16]. Nevertheless, ongoing study is needed to investigate the other protective mechanisms during CuONPs exposure.

## Conclusion

In summary, the most important discovery of the current study is demonstrating that the loss of *lc3b* will result in aggravated acute lung injury after CuONPs instillation, and highlights the protective role of autophagy upon CuONPs treatment. Autophagy deficiency seems to increase the copper ions due to the retardation of elimination of mitochondria overloaded with copper ions. These findings will provide a meaningful insight that targeting autophagy may serve as a valuable strategy for the treatment of CuONPs-associated toxicity.

## Materials and methods

### Chemicals and reagents

The following antibodies against GCLM (1:2000; ab126704), p62 (1:2000; ab109012), ATG5 (1:3000; ab109409), ATG7 (1:10,000; ab52472), LC3B (1:3000; ab192890), p-mTOR (1:2000; ab109268) were purchased from Abcam (Cambridge, UK). The following antibodies against BIP (1:1000; 3177), mTOR (1:1000; 2983), p-AMPK α (1:2000; 2573S), AMPKα (1:2000; 5831), Beclin1 (1:1000; 3495) were purchased from Cell Signaling Technology (Danvers, MA). The CD68 (1:200; 28058-1-AP) and HO-1 (1:1000; 10701-1AP) was purchased from Proteintech (Chicago, USA). The ULK1 (1:1000; A5149) was purchased from Bimake (Houston, USA). The β-actin Ab was obtained from Bioss (1:5000; bs-0061R) (Beijing, China). The QuantiChrom^TM^ Copper Assay Kit (DICU-250) was purchased from Bioassay (California, USA).

### Characterization and preparation of CuONPs

Characterization and preparation of CuONPs were performed according to our previous studies [35, 36]. The morphology of CuONPs was observed with transmission electron microscope (TEM) (Hitachi-7500; Hitachi, Ltd, Tokyo, Japan). The chemical elemental composition of this CuONPs was detected by field emission scanning electron microscopy (Hitachi SU8010) with energy-dispersive spectroscopy (Oxford X-MAN 50) (FE-SEM/EDS). The hydrodynamic diameter and zeta-potential of CuONPs were determined by dynamic light scattering (DLS) with Malvern Zetasizer Nano ZEN3690 (Malvern, Massachusetts, USA). The characteristics of CuONPs used in this study were presented in Fig. 1. For preparation of CuONPs, the CuONPs were firstly diluted to the concentration of 2 mg/mL. The suspension dilution is composed of 2% heat-inactivated sibling mouse serum in MilliQ water. Then the CuONPs suspension dilution was sonicated according to sonication procedure that 20% of maximum amplitude applied for 20 min on ice with an ultrasonic cleaner. To avoid the dissolution of CuONPs in suspension solution, the CuONPs were freshly prepared before administration.

### Animals and treatment

This study has been officially approved by Chongqing Medical University and a proof/certificate of approval is available upon request. C57BL/6J background *lc3b*^-/-^ mice were originally obtained from the Jackson Laboratory and their corresponding age and weight-matched wild-type C57BL/6J male mice were purchased from the Experimental Animal Center of Chongqing Medical University (Animal certificate number: SCXK, Chongqing, 2012-0001). The animals were randomly divided into the following 4 groups: Vehicle group, 1.5 mg/kg CuONPs group, 2.5 mg/kg CuONPs group and 5 mg/kg CuONPs group [17]. The illustration of the study design is shown in Additional file 1: Figure S1. The animals were kept in standard laboratory polycarbonate cages in controlled ambient temperature at 23 ℃ ± 1 ℃ with relative humidity of 50% ± 10%, and a light-dark cycle of 12/12 h. The mice had free access to standardized pellet food and tap water. The mice were exposed to CuONPs via intratracheal instillation after the habituation for seven days, and the treated mice were sacrificed 3 days after CuONPs treatment.

### Cell count, protein concentration and lactate dehydrogenase (LDH) in bronchoalveolar lavage fluid (BALF)

After the instillation of vehicle solution or CuONPs suspension, BALFs were obtained from the trachea by rinsing the lungs three times with warmed 0.9% sterile physiological saline. Then the BALFs were centrifuged at 2500 rpm for 10 min at 4 ℃. The pellets were collected and re-suspended with PBS, the cell counts in BALFs were further determined by TC20^TM^ Automated Cell Counter (Bio-Rad,Hercules, USA). The concentrations of protein in the supernatant without cells were quantified by BCA kits (Bio-Rad). Lactate dehydrogenase (LDH) activity in BALFs were detected by LDH assay kits according to the manufacturer’s protocol (NanJingJianCheng, Nanjing, China).

### Hematoxylin and eosin staining

After indicated treatment, the left lungs of mice were obtained by operation and quickly submerged in freshly prepared 4% paraformaldehyde/PBS (pH 7.4) for tissue fixation for 48 h at room temperature. Then the lungs were embedded in paraffin, cut into 5 μm sections, and stained with hematoxylin and eosin as described before [36]. Due to the patchy nature of acute lung injury induced by CuONPs, 30 random high-power fields (400X total magnification) were independently scored in a blinded fashion for each condition. The selection of random fields typically involves successive random displacements (each at least one high-power field in length) from the current position. To generate a lung injury score, the sum of each of the five independent variables, including neutrophils in the alveolar space, neutrophils in the interstitial space, hyaline membranes, proteinaceous debris filling the airspaces, and alveolar septal thickening, are weighted according to the relevance ascribed to each feature, and then were normalized to the number of fields evaluated. The resulting injury score was a continuous value between zero and one (inclusive) [37].

### Immunohistochemical analyses

The lungs of mice were embedded in paraffin and cut into 5 μm sections slides. Then, section slides were submerged in citric acid buffer and microwaved for 10 min to expose the antigens. 3% hydrogen peroxide was used to quench endogenous peroxidase. 10% goat serum was used to reduce nonspecific binding. Section slides were incubated with antibodies against CD68 (1:100) following incubation with corresponding second antibody. 3,3′-diaminobenzidine (DAB) based methods was used to observe CD68-postive cells in section slides under a fluorescence microscope (Olympus IX53, Tokyo, Japan) as described before [36]. The numbers of CD68- positive cells were counted by using Image J software to calculate the intensity of CD68.

### Transmission electron microscope

Ultrastructure of mice lung tissues in indicated groups were detected by using JEM-1400 plus transmission electron microscope (JEOL, Peabody, MA, USA) as described before [36]. In brief, the mice were sacrificed to obtain lung tissues. Then about 1 mm^3^ volume of lung tissues were cut by sharp blade and quickly submerged in 2.5% glutaraldehyde solution at 4 °C for at least 24 h for stable fixation. Subsequently, the section slides were rinsed and further fixed in osmic acid. After dehydration with alcohol and acetone gradient, the blocks were embedded with epoxy resin. 1 μm ultrathin section were obtained and further stained with saturated acetate uranium.

### Copper ions content in lung tissues

The concentrations of free copper ions in mice lung tissues were determined by QuantiChrom Copper Assay Kit (BioAssay Systems, Hayward, CA, USA) as described previously [7]. In brief, the supernatants of lung tissues of mice with or without CuONPs treatment were collected. Reagent A was mixed with the above supernatants. After centrifugation with 16,000 × g for 2 min, the 100 μL clear supernatant was obtained and was ready for assay. Working reagent is composed of Reagent B and Reagent C. After thoroughly mixture of supernatant and working reagent for 5 min at room temperature, the absorbance of each well was measured by VERS Amax Microplate Reader (Molecular Devices Corp, Sunnyvale, CA, USA). The concentrations of copper ions in mice lung tissues were calculated according to the obtained absorbance.

### Western blotting

Western blotting was performed according to our previous study [38]. A total of 20–40 μg of protein extracted from lung tissues of mice with indicated treatment was loaded into tris-glycine SDS-PAGE gels and separated at 80 V. The protein in gels were subsequently transferred onto the PVDF membrane (Millipore). After blocking with 5% nonfat milk in TBST, the membrane were incubated with indicated antibodies against indicated protein overnight at 4 °C followed incubation with HRP-conjugated secondary antibodies. The signal of indicated protein was detected using the ChemiDoc Touch Imaging system (1708370, Bio-Rad).

### Quantitative PCR

The total RNA from lung tissues was obtained using the TRizol reagent (KeyGen BioTECH, Nanjing, China). Then the complementary DNA (cDNA) was synthesized by T100 Thermal Cycler (Bio-Rad). The expressions of target genes were assessed by CFX Connect™ Real-Time PCR Detection System (Bio-Rad). TBP was used as internal control gene. The sequences of primer were included in Table 1.

**Table 1:** QPCR primers used in this study

Gene names	Forward primer (5′-3′)	Reverse primer (5′-3’)
*Il-6*	CATCCAGTTGCCTTCTTG	ATTAAGCCTCCGACTTGT
*Il-1β*	GGACAGAATATCAACCAACAA	TTACACAGGACAGGTATAGATT
*Tnf-α*	TCTCAGCCTCTTCTCATTC	GCCATTTGGGAACTTCTC
*Ccl2*	ATGAGATCAGAACCTACAACT	TCCTACAGAAGTGCTTGAG
*Sele*	AATGAGGACTGTGTAGAGATT	GCAGGTGTAACTATTGATGG
*Nfe2l2*	CAGCATAGAGCAGGACAT	GGAACAGCGGTAGTATCA
*Hspa5*	AGCCAACTGTAACAATCAAG	GTCACTCGGAGAATACCAT
*Hmox1*	AATGAACACTCTGGAGATGA	CAATGTTGAGCAGGAAGG
*Gclm*	GATGCCACCAGATTTGAC	CTTCACGATGACCGAGTA
*Tbp*	CCTTCACCAATGACTCCTA	CAAGATTCACGGTAGATACAAT

### Statistical analysis

The results were presented as mean ± standard deviation (S.D.) and the experiments were performed at least 3 times independently. One-way analysis of variance (ANOVA) with the least significant difference *t*-test or independent student *t*-test was used to assess the significant difference. Data analysis were performed with Graphpad prism software (San Diego, CA, USA). The statistical significance level was set at *p* value less than 0.05.

## Supplementary Information

**Additional file1: Figure S1.** Scheme of study design. C57BL/6J mice were exposed to vehicle, 1.5, 2.5 or 5 mg/kg CuONPs, respectively. After 3 days, the lung tissues and BALF were collected for lung injury assessment. To determine the role of autophagy in lung injury-induced by CuONPs, wild type and *lc3b *knockout mice were exposed to vehicle or 5 mg/kg CuONPs, respectively. After 3 days, the lung tissues and BALF were collected for lung injury assessment.

## Data Availability

The authors declare that the data related to this study are provided upon request.

## References

[1] Verma N, Kumar N (2019). Synthesis and biomedical applications of copper oxide nanoparticles: an expanding horizon. ACS Biomater Sci Eng.

[2] Meghana S, Kabra P, Chakraborty S, Padmavathy N (2015). Understanding the pathway of antibacterial activity of copper oxide nanoparticles. RSC Adv.

[3] Wang Y, Yang F, Zhang HX, Zi XY, Pan XH, Chen F, Luo WD, Li JX, Zhu HY, Hu YP (2013). Cuprous oxide nanoparticles inhibit the growth and metastasis of melanoma by targeting mitochondria. Cell Death Dis.

[4] Yang Q, Wang Y, Yang Q, Gao Y, Duan X, Fu Q, Chu C, Pan X, Cui X, Sun Y (2017). Cuprous oxide nanoparticles trigger ER stress-induced apoptosis by regulating copper trafficking and overcoming resistance to sunitinib therapy in renal cancer. Biomaterials.

[5] Ivask A, Titma T, Visnapuu M, Vija H, Kakinen A, Sihtmae M, Pokhrel S, Madler L, Heinlaan M, Kisand V (2015). Toxicity of 11 metal oxide nanoparticles to three mammalian cell types in vitro. Curr Top Med Chem.

[6] Cronholm P, Karlsson HL, Hedberg J, Lowe TA, Winnberg L, Elihn K, Wallinder IO, Moller L (2013). Intracellular uptake and toxicity of Ag and CuO nanoparticles: a comparison between nanoparticles and their corresponding metal ions. Small.

[7] Zhang J, Zou Z, Wang B, Xu G, Wu Q, Zhang Y, Yuan Z, Yang X, Yu C (2018). Lysosomal deposition of copper oxide nanoparticles triggers HUVEC cells death. Biomaterials.

[8] Zhang J, Wang B, Wang H, He H, Wu Q, Qin X, Yang X, Chen L, Xu G, Yuan Z (2018). Disruption of the superoxide anions-mitophagy regulation axis mediates copper oxide nanoparticles-induced vascular endothelial cell death. Free Radic Biol Med.

[9] He H, Zou Z, Wang B, Xu G, Chen C, Qin X, Yu C, Zhang J (2020). Copper oxide nanoparticles induce oxidative dna damage and cell death via copper ion-mediated p38 mapk activation in vascular endothelial cells. Int J Nanomedicine.

[10] Sun T, Yan Y, Zhao Y, Guo F, Jiang C (2012). Copper oxide nanoparticles induce autophagic cell death in A549 cells. PLoS One.

[11] Orrenius S (2007). Reactive oxygen species in mitochondria-mediated cell death. Drug Metab Rev.

[12] Baker ZN, Cobine PA, Leary SC (2017). The mitochondrion: a central architect of copper homeostasis. Metallomics.

[13] Stern ST, Adiseshaiah PP, Crist RM (2012). Autophagy and lysosomal dysfunction as emerging mechanisms of nanomaterial toxicity. Part Fibre Toxicol.

[14] Wei F, Duan Y (2019). Crosstalk between autophagy and nanomaterials: internalization, activation termination. Adv Biosyst.

[15] Jiang Y-W, Gao G, Jia H-R, Zhang X, Zhao J, Ma N, Liu J-B, Liu P, Wu F-G (2019). Copper oxide nanoparticles induce enhanced radiosensitizing effect via destructive autophagy. ACS Biomater Sci Eng.

[16] Gosens I, Cassee FR, Zanella M, Manodori L, Brunelli A, Costa AL, Bokkers BG, de Jong WH, Brown D, Hristozov D (2016). Organ burden and pulmonary toxicity of nano-sized copper (II) oxide particles after short-term inhalation exposure. Nanotoxicology.

[17] Lai X, Zhao H, Zhang Y, Guo K, Xu Y, Chen S, Zhang J (2018). Intranasal delivery of copper oxide nanoparticles induces pulmonary toxicity and fibrosis in C57BL/6 mice. Sci Rep.

[18] Maes M, Vanhaecke T, Cogliati B, Yanguas SC, Willebrords J, Rogiers V, Vinken M (2015). Measurement of apoptotic and necrotic cell death in primary hepatocyte cultures. Methods Mol Biol.

[19] Kapoor M, Martel-Pelletier J, Lajeunesse D, Pelletier JP, Fahmi H (2011). Role of proinflammatory cytokines in the pathophysiology of osteoarthritis. Nat Rev Rheumatol.

[20] Turner MD, Nedjai B, Hurst T, Pennington DJ (2014). Cytokines and chemokines: at the crossroads of cell signalling and inflammatory disease. Biochim Biophys Acta.

[21] Farrell HE, Lawler C, Oliveira MT, Davis-Poynter N, Stevenson PG (2015). Alveolar macrophages are a prominent but nonessential target for murine cytomegalovirus infecting the lungs. J Virol.

[22] Baggiolini M (1998). Chemokines and leukocyte traffic. Nature.

[23] Silva M, Videira PA, Sackstein R (1878). E-selectin ligands in the human mononuclear phagocyte system: implications for infection, inflammation, and immunotherapy. Front Immunol.

[24] Chen ZH, Wu YF, Wang PL, Wu YP, Li ZY, Zhao Y, Zhou JS, Zhu C, Cao C, Mao YY (2016). Autophagy is essential for ultrafine particle-induced inflammation and mucus hyperproduction in airway epithelium. Autophagy.

[25] Li Y, Ju D (2018). The role of autophagy in nanoparticles-induced toxicity and its related cellular and molecular mechanisms. Adv Exp Med Biol.

[26] Mohammadinejad R, Moosavi MA, Tavakol S, Vardar DO, Hosseini A, Rahmati M, Dini L, Hussain S, Mandegary A, Klionsky DJ (2019). Necrotic, apoptotic and autophagic cell fates triggered by nanoparticles. Autophagy.

[27] Luzio JP, Pryor PR, Bright NA (2007). Lysosomes: fusion and function. Nat Rev Mol Cell Biol.

[28] Laha D, Pramanik A, Maity J, Mukherjee A, Pramanik P, Laskar A, Karmakar P (2014). Interplay between autophagy and apoptosis mediated by copper oxide nanoparticles in human breast cancer cells MCF7. Biochim Biophys Acta.

[29] Cho WS, Duffin R, Poland CA, Duschl A, Oostingh GJ, Macnee W, Bradley M, Megson IL, Donaldson K (2012). Differential pro-inflammatory effects of metal oxide nanoparticles and their soluble ions in vitro and in vivo; zinc and copper nanoparticles, but not their ions, recruit eosinophils to the lungs. Nanotoxicology.

[30] Ahamed M, Akhtar MJ, Alhadlaq HA, Alrokayan SA (2015). Assessment of the lung toxicity of copper oxide nanoparticles: current status. Nanomedicine.

[31] Kim J, Kundu M, Viollet B, Guan KL (2011). AMPK and mTOR regulate autophagy through direct phosphorylation of Ulk1. Nat Cell Biol.

[32] Leary SC, Winge DR, Cobine PA (2009). "Pulling the plug" on cellular copper: the role of mitochondria in copper export. Biochim Biophys Acta.

[33] Cobine PA, Ojeda LD, Rigby KM, Winge DR (2004). Yeast contain a non-proteinaceous pool of copper in the mitochondrial matrix. J Biol Chem.

[34] Bhattacharjee A, Yang H, Duffy M, Robinson E, Conrad-Antoville A, Lu YW, Capps T, Braiterman L, Wolfgang M, Murphy MP (2016). The activity of Menkes disease protein ATP7A is essential for redox balance in mitochondria. J Biol Chem.

[35] Zhang S, Jiang X, Cheng S, Fan J, Qin X, Wang T, Zhang Y, Zhang J, Qiu Y, Qiu J (2020). Titanium dioxide nanoparticles via oral exposure leads to adverse disturbance of gut microecology and locomotor activity in adult mice. Arch Toxicol.

[36] Jiang X, Tang Q, Zhang J, Wang H, Bai L, Meng P, Qin X, Xu G, Bose DD, Wang B (2018). Autophagy-dependent release of zinc ions is critical for acute lung injury triggered by zinc oxide nanoparticles. Nanotoxicology.

[37] Matute-Bello G, Downey G, Moore BB, Groshong SD, Matthay MA, Slutsky AS, Kuebler WM (2011). Acute lung injury in animals study G: an official American Thoracic Society workshop report: features and measurements of experimental acute lung injury in animals. Am J Respir Cell Mol Biol.

[38] Zhang J, Qin X, Wang B, Xu G, Qin Z, Wang J, Wu L, Ju X, Bose DD, Qiu F (2017). Zinc oxide nanoparticles harness autophagy to induce cell death in lung epithelial cells. Cell Death Dis.

