# Supplementary material for: Autophagy deficiency exacerbates acute lung injury induced by copper oxide nanoparticles
Source: J Nanobiotechnology. 2021 May 31;19:162. doi: 10.1186/s12951-021-00909-1 (PMC8166141; doi:10.1186/s12951-021-00909-1)
Supplement: Supplementary file 1 — Additional file1: Figure S1. Scheme of study design. C57BL/6J mice were exposed to vehicle, 1.5, 2.5 or 5 mg/kg CuONPs, respectively. After 3 days, the lung tissues and BALF were collected for lung injury assessment. To determine the role of autophagy in lung injury-induced by CuONPs, wild type and lc3b knockout mice were exposed to vehicle or 5 mg/kg CuONPs, respectively. After 3 days, the lung tissues and BALF were collected for lung injury assessment. [file 12951_2021_909_MOESM1_ESM.docx]

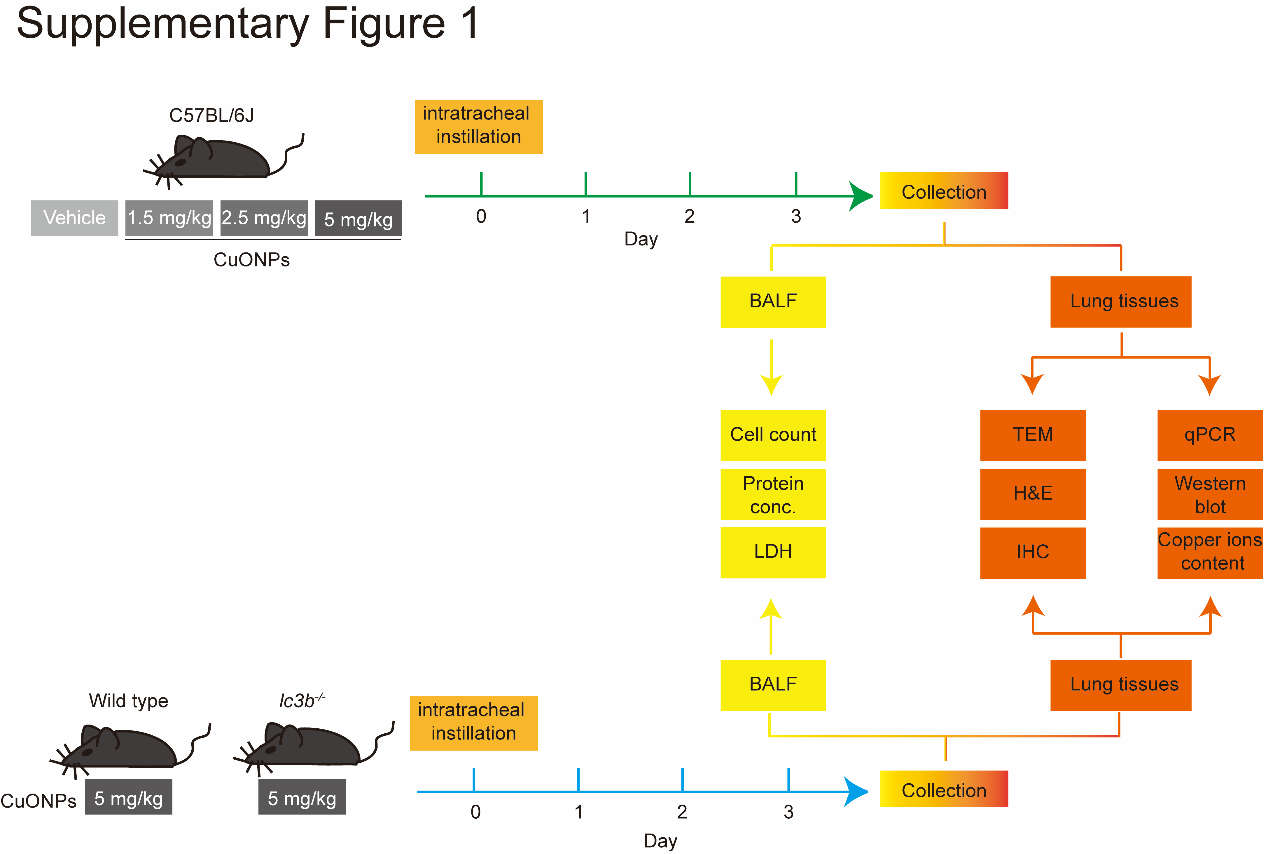


**Supplementary Figure 1. Scheme of study design.**

C57BL/6J mice were exposed to vehicle, 1.5, 2.5 or 5 mg/kg CuONPs, respectively. After 3 days, the lung tissues and BALF were collected for lung injury assessment. To determine the role of autophagy in lung injury-induced by CuONPs, wild type and *lc3b* knockout mice were exposed to vehicle or 5 mg/kg CuONPs, respectively. After 3 days, the lung tissues and BALF were collected for lung injury assessment.
